# Understanding Keloid Pathobiology From a Quasi-Neoplastic Perspective: Less of a Scar and More of a Chronic Inflammatory Disease With Cancer-Like Tendencies

**DOI:** 10.3389/fimmu.2019.01810

**Published:** 2019-08-07

**Authors:** Silvian Tan, Nonhlanhla Khumalo, Ardeshir Bayat

**Affiliations:** ^1^Plastic and Reconstructive Surgery Research, Centre for Dermatology Research, NIHR Manchester Biomedical Research Centre, University of Manchester, Manchester, United Kingdom; ^2^Hair and Skin Research Laboratory, Department of Dermatology, Groote Schuur Hospital, University of Cape Town, Cape Town, South Africa

**Keywords:** keloid, skin scarring, quasi-neoplastic, tumor, cancer, bioenergetics, fibroproliferative, epigenetics

## Abstract

Keloids are considered as benign fibroproliferative skin tumors growing beyond the site of the original dermal injury. Although traditionally viewed as a form of skin scarring, keloids display many cancer-like characteristics such as progressive uncontrolled growth, lack of spontaneous regression and extremely high rates of recurrence. Phenotypically, keloids are consistent with non-malignant dermal tumors that are due to the excessive overproduction of collagen which never metastasize. Within the remit of keloid pathobiology, there is increasing evidence for the various interplay of neoplastic-promoting and suppressing factors, which may explain its aggressive clinical behavior. Amongst the most compelling parallels between keloids and cancer are their shared cellular bioenergetics, epigenetic methylation profiles and epithelial-to-mesenchymal transition amongst other disease biological (genotypic and phenotypic) behaviors. This review explores the quasi-neoplastic or cancer-like properties of keloids and highlights areas for future study.

## Introduction

Keloids are considered as benign fibroproliferative dermal tumors, which are borne out of abnormal wound healing processes following injury to the skin. They are characterized visually by raised exophytic dermal outgrowths extending beyond the original wound boundary and microscopically by thickened hyalinized collagen bundles (1, 2).

Most individuals affected by keloid disease are aged between 10 and 30 years and are of pigmented skin with high reports in African, Afro-Caribbean, Afro-American, Hispanic, or Asian ancestry (3–6). Keloids may develop months or years after the initial injury and can be accompanied by intense pain, pruritus, and other physical, and psychosocial symptoms (7). Common sites affected are the anterior chest, shoulders, back, and earlobe, with those on the pre-sternum and shoulder regions developing under high tension. Keloids tend to be aggressive in invading adjacent surrounding healthy (normal) skin and can often recur following any form of treatment. In particular, monotherapy with surgical removal alone carries a recurrence rate of up to 100% (8). Phenotypically, keloids are consistent with non-malignant dermal tumors that are due to the excessive overproduction of collagen which never metastasize. However, the morphology and clinically aggressive behavior of keloids can be thought to bear a resemblance to neoplastic dermal tumors.

Different theories exist to explain the etiology of keloids, including elevated skin tension (9, 10), hypoxia (11), chronic inflammation (12), autoimmune (13–15), genetics (16, 17), and vascular factors (18), none of which, however, are independently sufficient to do so. To date, options for keloid treatment are poorly defined, in part due to unsatisfactory outcomes of current treatments and the poor quality of evidence surrounding their use. Compounding keloid research is the lack of animal disease models for testing.

**Figure 1:**
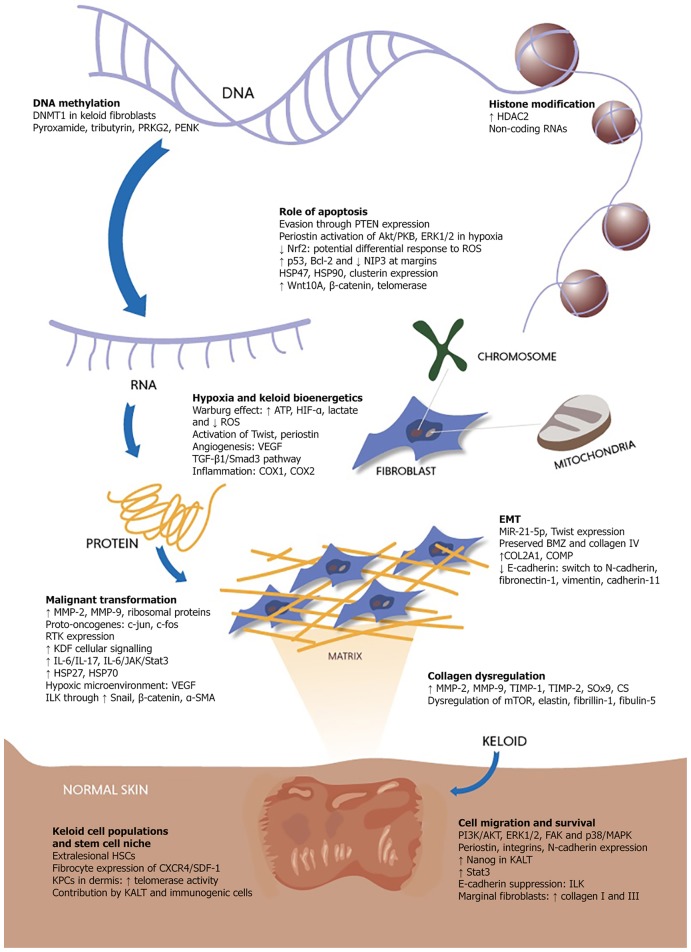
Key processes contributing to the quasi-neoplastic expression of keloid pathobiology.

This review aims to explore the salient cancer-like or quasi-neoplastic attributes and features of keloids (Figure 1) and highlights several key areas for future study.

## Keloids: Scars or Cancerous Tumors?

Keloids are traditionally viewed as scars on the spectrum of fibroproliferative dermal diseases. To elucidate the etiopathogenesis of keloids, however, the distinction between scar and disease must be made. A scar is the endpoint of physiological wound healing, preceded by inflammation, fibroplasia and granulation, and manifest as non-functioning fibrotic tissue, which may regress over time (19, 20). Cutaneous scars often undergo changes in properties such as thickness, texture, color, and strength but remain within the confines of the surrounding intact skin (19, 21). In contrast, keloids are aggressive exophytic dermal outgrowths disproportionately grown beyond the boundaries of the original wound, from a source, which remains suspended in “wound healing” and scar maturation. Although keloids are not routinely classified as true neoplasms due to their lack of spontaneous occurrence and absence of metastasis, they exhibit various cancer-like characteristics such as uncontrolled proliferation, invasiveness into surrounding tissue, lack of spontaneous regression and ability to vascularize (22–24). Their disproportionately locally aggressive clinical phenotype suggests possible links with skin or mesenchymal tumors that need to be explored in further detail.

Owing to their similar clinical presentation, keloids have been misdiagnosed as other benign and malignant skin tumors and vice versa (25). Of these, dermatofibrosarcoma protuberans is the most commonly linked to keloids amongst others such as keloidal dermatofibroma (26), keloidal basal cell carcinoma, keloidal atypical fibroxanthoma (27), suggesting a possible overlap between them (28). Based on their pathobiology and clinical phenotype, keloids bear the most similarity between tumors relating to fibrosis, dermal origin, and mesenchymal origin (29). Similarities between keloids and mesenchymal tumors can be found across a range of characteristics from biomarker expression to clinical presentation (30). Seminal work on keloid mesenchymal-related biomarkers suggests that stem-cell like cells identified in keloids deviate from dermal to chondrocytic or osteogenic lineage (31), however research to confirm such hypotheses in this direction is still lacking.

## Keloid Morphology: Cellular and Matrix Composition

On gross examination, keloids are well-demarcated, raised cutaneous lesions in different shapes and may be bosselated, nodular, or pendulous (32). They may appear shiny with a discoloration—often erythematous with telangiectasia in individuals with fairer skin and hyperpigmented in those with darker skin (32)—and can reach sizes between several millimeters to many centimeters in diameter (33). Keloids display a heterogenic phenotype in relation to stiffness and range from soft to extremely firm with decreased skin plasticity. The center of keloid tissue often exhibit central hypoxia due to capillary occlusion as a result of exuberant collagen and endothelial cells (34). Keloid margins contain active fibroblasts which invade into surrounding tissue and are well-vascularized through angiogenesis to upkeep the oxygen and nutrient supply required presumably to fuel their invasiveness (24, 35). This is thought to be the result of endothelial cell migration and survival from growth factor activation through kinase signaling pathways such as PI3K/AKT, ERK1/2, FAK, and p38/MAPK (36, 37).

Most of the expansile keloid tissue is in the reticular dermal layer with a thickened overlying epidermis. Keloid tissues are not encapsulated and have a perimeter that advances into the surrounding tissue (2). Marginal fibroblasts are more metabolically active and engage in higher rates of collagen I and III production, as reflected by more erythematous skin overlying these margins (38). Several studies have shown that the outer margins and inner complex of keloids are populated by different types of stem cells. In particular, mesenchymal-like stem cells expressing non-hemapoetic markers constituting the inner complex appear to represent the niche that is vital to sustain keloid growth, surrounded by extralesional hematopoietic stem cells (39, 40). In relation to this, the concentration of homogenous thickened hyalinized collagen in the center surrounded by more typical-looking collagen fibers near the edges is unique to keloids (41), but not always present (2).

In brief, cutaneous wound healing involves the formation and remodeling of collagen matrix over time by fibroblasts and myofibroblasts. Similarly, activated myofibroblasts and keloid fibroblasts represent the main generators of keloid matrix through their synergistic action in increasing keloid tissue stiffness (42, 43). Fibrocytes, thought to be myofibroblast precursors, are bone marrow-derived circulating cells present in keloids and tissues undergoing wound healing (44–46). They appear as a cross between fibroblasts, monocytes, and hematopoietic stem cells (HSCs) due to their fibroblast products, hematopoietic surface markers, myeloid antigen expression, and shared morphological characteristics (47). Their expression of *CXCR4* forms part of the CXCR4/SDF-1 axis, which is crucial in skin regulating cutaneous wound healing, systematic lupus erythematosus and angiogenesis of basal cell carcinoma (48).

The keloid matrix consists of different collagens, glycoproteins, and glycosaminoglycans (GAGs) (23, 49, 50). The initial overproduction of type III collagen in keloid matrix is replaced by type I collagen, with an extremely high ratio of type I to type III collagen (17:1) compared to normal scars (6:1) (51). The collagen fibers constituting keloids are larger than those in normal scars and disorderly arranged (2) in loosely cross-linked (51) whorls of thick bundles in the same plane as the epidermis (1). Elevated levels of *MMP-2, TIMP-1*, and *TIMP-2* are observed in keloids (52), the imbalance of which appears to dysregulate collagen production and accumulation, with upregulated *MMP-2* and *MMP-9* expression additionally linked to cancer invasiveness (53). Overproduction of collagen and matrix is also linked with dysregulated *mTOR* signaling (54), which plays a crucial role in human cancers (55). Interestingly, elastic fiber deposition is higher in the reticular dermis than the papillary dermis (56, 57), but has been shown to be absent in the keloid extracellular matrix (ECM) alongside deregulated expression of elastic fiber assembly proteins such as elastin, fibrillin-1 and fibulin-5 (58). This is attributed to the over-deposition of chondroitin sulfate (CS) which is 6.9 times higher than that in normal skin. CS is thought to suppress elastic fiber assembly through the dysregulation of fibrillin-1 deposition (58) and its aberrant expression has been found to contribute to tumor metastasis in breast cancer (59). The functions of fibulin-5 and elastin in tumor formation are complex, involving the regulation of metalloproteinases (MMPs) amongst others (60–62). The interplay between these proteoglycan-associated factors in keloid pathogenesis remain to be elucidated.

*Sox9*, the master regulator of chondrogenesis (63), is upregulated in keloids (31) and its ectopic expression is linked with the upregulation of *COL2A1* and cartilage oligomeric matrix protein (*COMP*) which culminates in ECM production geared toward chondrogenesis and fibrosis (64). *COMP* stimulates the assembly of collagen 1 fibrils (65, 66), with an expression level proportional to keloid size and is expressed in scleroderma and other tumors (67–69). Keloids exhibit significantly raised levels of biglycan in the nodular dermis of active keloid lesions alongside decreased decorin expression (70). This is interesting as decorin, which has recently been found to suppress collagen production, is raised in malignant conditions (71, 72). All these proteoglycans have been shown to be differentially regulated by basic fibroblast growth factor, that is thought to be involved in producing the tumor phenotype (73, 74).

Keloids share many similarities with other dermal tumors by virtue of their shared tissue origin. This includes the integral activation of Wnt β-catenin pathway in desmoid tumors (75) and elevated levels of TGF-β, collagen, and GAG in dermatofibrosarcoma protuberans (76). In both of these tumors, trauma has been cited as a precipitating factor (75, 77).

## Keloids and the Hallmarks of Cancer

Although keloids exhibit the hallmarks of cancer to a large extent, these characteristics remain to be fully explored in this context (78, 79). There is potentially a key regulator or group of pre-requisite factors, which when activated after skin injury, triggers a cascade of events that culminate in keloid scar formation. The various relationships between tumor-related factors expressed in keloids may be complex and their roles in sustaining keloid growth are still unclear. Table 1 highlights key tumor biomarkers discussed in this review.

**Table 1:** Expression of tumor biomarkers in keloids.

**Biomarker**	**Normal physiological function**	**Associated tumor processes**	**Expression in keloids**	**References**
Bcl-2	Apoptosis regulation	Dysregulated in skin cancers and upregulated in some chondrosarcomas	Positive in basal keratinocytes and spindle-shaped cells	(79–82)
BMP2	TGF-β family	Highly expressed in malignant fibrous histiocytoma	↑	(31, 83)
c-jun	Role in organogenesis	Proto-oncogene which promotes cell migration and motility factor ENPP2 in soft tissue sarcomas and is strongly expressed in BCC	Expressed in keloid keratinocytes but absent in resting keloids	(82, 84–86)
CXCR4	Increases fibroblastic expression of collagen and TGF-β	Cross-talk between tumor cells and tumor micro-environment	Positive	(87, 88)
HIF-1	Response to hypoxia	Adaptation of tumor cells to hypoxia	↑	(89, 90)
HSP27	Stress response protein	Highly expressed in cancers	↑	(91–93)
HSP47	Collagen maturation	Promotes cell survival in various mesenchymal tumors	↑	(94–96)
HSP90	Indicates cellular replicative potential		↑	(97, 98)
IL-6	Interleukin with pro- and anti-inflammatory roles	Pro-tumorigenic	↑	(39, 99, 100)
SERPINB5	P53-regulated tumor suppressor	Expressed in various cancers	Positive	(101–103)
MMP-2, MMP-9	ECM degradation	Expressed in various cancers	↑	(53, 104–108)
MRF 15	Cell senescence	Senescence induction of human tumor cell lines	↑ in keloid fibroblasts	(23, 109)
mTOR	Cell signaling mediator	Dysregulated in various cancers	↑	(55, 110)
Nanog	ESC marker	Multifaceted role in cancers, positive in bone sarcomas	↑ in KPCs, KALT	(39, 111–113)
NIP3	Pro-apoptotic, induced in response to hypoxia	Expressed in various tumors	↓	(114–116)
Notch	Cell-to-cell signaling	Dual role in tumor mediation as promoter and suppressor	↑	(117, 118)
Oct4	ESC marker	Regulates EMT and ERK1/2 signaling in ovarian cancer, positive in bone sarcomas	↑ in KPCs, KALT	(39, 112, 113, 119)
Osteopontin	Regulation of cellular functions	Promotes invasiveness in various carcinomas	Expressed in epidermis	(120, 121)
p53	Tumor suppressor gene	Mutated in more than 50% of all tumors	↑ in central region of KFs	(122, 123)
p63	Inhibits p53 translational activation	Expressed in many benign giant cell bone tumors and uncommon in malignant tumors	↑	(124–126)
PDGF	Protein tyrosine kinase (PTK)	Mutated in some cancers	Positive	(99, 100, 127)
Periostin	Collagen fibrillogenesis	Tumor progression and metastasis	↑	(31, 128, 129)
ROS	Apoptosis induction in DNA damage	Second messenger in intracellular signaling in cancer	↑	(130, 131)
S100	Upregulated in skin tumors	Elevated in osteosarcoma	Variable expression	(132, 133)
Sox2	ESC marker	Tumor initiation and maintenance of CSCs in osteosarcomas and squamous cell carcinomas	↑	(112, 134, 135)
Sox9	Master regulator of chondrogenesis	Present in classic and mesenchymal chondrosarcoma	↑	(31, 63, 136)
STAT3	ESC marker and oncogene activated by PDGF and IL-6	Activated in various cancers and tumor cell lines	Expressed in endothelium of KALT microvessels	(112, 137–139)
Wnt10α	Anti-apoptosis	Promotes carcinogenesis	↑	(140–142)

### Sustaining Proliferative Signaling

Keloid fibroblasts express higher receptor tyrosine kinase signals compared to normal skin derived fibroblasts (143, 144). This increased keloid-derived fibroblast cellular signaling, which may influence cell growth, differentiation, and survival, is linked to cancer development when dysregulated (145). *Nanog*, a gene which confers self-replication abilities to cells and is elevated in various cancers (111, 146, 147), is absent in somatic cells (148), yet recently found to be upregulated in keloid associated lymphoid tissue (KALT) (112). Zhang et al. (39) described a population of clonogenic, self-renewing keloid-derived precursor cells expressing mesenchymal and embryonic stem cell markers and are driven by IL-6/IL-17 mediated inflammation. IL-6, alongside hepatocyte growth factor and epidermal growth factor, is pro-tumorigenic (39). Of interest, IL-6 and its second messengers JAK/Stat3, both of which are elevated in keloid fibroblasts, form the IL-6/JAK/Stat3 pathway, which contributes toward tumorigenesis within the tumor micro-environment. In particular, Stat3 confers increased cell proliferation and migration within keloid fibroblasts (99, 149).

Transforming growth factor beta (TGF-β) is a well-researched cytokine in keloid pathogenesis due to its pivotal role in directing keloid fibrosis (150, 151). TGF-β1 influences keloid keratinocytes through both Smad-dependent and Smad-independent signaling pathways such as ERK1/2 and p38 to promote collagen accumulation (150, 151). In relation to cancer, TGF-β1 has been shown to inhibit initial tumor formation before accelerating its malignancy in a transgenic mouse skin cancer model (152). Keloid and normal fibroblasts exhibit a differential response to PDGF, EGF, FGF (153), and TGF-β (154), the majority of which bind to protein tyrosine kinase (PTK) family receptors. PTK receptors contribute to cell proliferation, differentiation (155), and carcinogenesis (156).

Satish et al. (23) found the upregulation of ribosomal proteins in keloid fibroblasts, which is also seen in cancer growth, indicating tighter regulations in keloids which prevent the malignant transformation of their precursor cells. Transcriptional activating factors involved include *c-jun* and *c-fos* which function as proto-oncogenes. In addition to its role in cell cycle regulation of fibroblasts (84) and Ras-activated human epidermal neoplasia, *c-jun* is also linked to benign inflammatory conditions like psoriasis and arthritis (157).

### Evasion of Growth Suppressors

The mechanisms underlying keloid growth are not straightforward, involving both up- and downregulation of tumor suppressors. The deregulation of *p53* forms the hallmark in various cancers; more recently a mechanism by which it is deregulated in keloids has been identified as TRAF4-USP10 interaction that culminates in fibroblast proliferation (158). Interestingly, whilst *p53* mutation is prevalent in keloids (159), its expression has also been found to be elevated in the central region of keloid fibroblasts alongside an increase in *p63* which inhibits its translation (101, 160). In turn, this leads to downstream effects including elevated *SERPINB5* and *maspin*, the latter of which is also upregulated during keratinocyte senescence (101). The link between TP53 codon 72 polymorphism and keloids remains unclear (124, 161, 162). Other examples include downregulated miR-1224-5p which normally functions through TGF-β1/Smad3, and has a role in cancers (163). Similarly, miR-21-5p plays a key role in PTEN-mediated proliferative and apoptotic mechanisms in keloid fibroblasts (164), causing decreased levels of *PTEN* which is a tumor suppressor with wide-ranging downstream effects on cellular maintenance and morphology through PI3K/AKT/mTOR (165). Interestingly, elevated levels of tumor suppressors such as *PML* are also seen in the hypercellular regions of keloids, suggesting senescence as a factor which confers keloids their benign nature (166). It is plausible that the concerted interplay between these factors result in the quasi-neoplastic behavior of keloids.

### Induction of Angiogenesis: The Role of Hypoxia in Keloid Bioenergetics

The Warburg effect is a phenomenon in cancer cells in which metabolism is favored and ATP is produced through anaerobic glycolysis in favor of oxidative metabolism. In cancer cells, the rate of glucose uptake is dramatically increased leading to lactate production, despite the presence of fully functioning mitochondria and availability of oxygen. Specifically, this refers to an increased rate of glycolysis, which is followed by a surge of lactic acid fermentation even if there is no lack of oxygen (167, 168). Similarly, keloid fibroblasts have been found to preferentially utilize the glycolytic pathway instead of oxidative phosphorylation, the latter of which is utilized by normal fibroblasts. The ability of keloid fibroblasts to tap into a wider source of energy may explain why keloids thrive in a hypoxic microenvironment. Indeed, the same study reported that keloid fibroblasts have more ATP, are 3.7 times more active than normal fibroblasts and exhibit lower levels of reactive oxygen species (ROS), which is a byproduct of mitochondrial oxidative phosphorylation (22). The switch to aerobic glycolysis in tumors as a means of ATP production is thought to be the result of glycolytic enzymes activated by HIF-α (169), which is also upregulated in keloids (170, 171). These findings are supported by the higher levels of lactate in keloids due to glycolysis and increase in keloid ATP synthesis on exposure to hypoxia (89, 172). As tumors become increasingly reliant on glycolysis with tumor progression (173), it may even be possible to correlate the growth of keloids with their utilization of glycolysis as a form of keloid disease staging.

Hypoxia is thought to promote tumor proliferation and invasiveness in cancer (174). In keloids, local hypoxia within the injury zone accelerates wound healing by stimulating angiogenesis and driving fibroblast proliferation (175), as evidenced by hypoxia-induced vascular endothelial growth factor (VEGF) expression (176) in keloid fibroblasts and a higher density of blood vessels in keloids than normal dermis and scars. In addition to angiogenesis, hypoxia-induced fibroblast to myofibroblast-like conversion has also been observed in keloids and postulated to occur by the TGF-β1/Smad3 pathway (177). Angiogenesis is vital in the development of many cutaneous diseases (178). Hypoxia induces the activation of HIF-α1 in both keloids and cancer (179). This results in the activation of Twist which mediates EMT-related cadherin switching and is a master regulator of morphogenesis and other embryonic processes (180).

Periostin, a cell-adhesion ECM protein (128), is increased in physiological processes involved in skin repair, fibrosis, cell proliferation, and ECM remodeling, and tumor processes such as growth and metastasis (181). Periostin increases angiogenesis by activating ERK1/2 and focal adhesion kinase (FAK) pathways, increasing VEGF and angiopoeitin-1 (Ang-1) expression (182). Specific to keloids, periostin is associated with promoting the ability of keloid fibroblasts to migrate and invade surrounding tissues in hypoxia (183). Periostin expression is upregulated by hypoxia through an HIF-1α-dependent pathway (183). Another group of cell adhesive receptor proteins implicated are integrins which are thought to interact with periostin to facilitate EMT, angiogenesis, and the mobility of chondrocytes, fibroblasts, and cancer cells (184). Periostin promotes angiogenesis in gastric, breast, and ovarian cancers (185, 186).

### Activating Invasion and Metastasis

There is a growing body of evidence recognizing the importance of EMT in keloid pathophysiology. Whilst EMT is known to promote the migratory behavior of metastatic cells, the benign nature of keloids makes it unclear whether they are the result of type II fibrotic EMT or suspended type III metastatic EMT (187). There is no evidence of significant epidermal-dermal basement membrane zone (BMZ) breakdown or disrupted collagen IV expression in the BMZ of keloids (188), which may explain why they do not metastasize. Investigating other cell motility factors in keloids may reveal unique key agents in metastatic prevention.

E-cadherin, a component of epithelial adherens junction, is encoded by *CDH1* and becomes lost in cancer cells undergoing EMT (189). Although *CDH1* gene expression levels are similar between normal and keloid keratinocytes, keloids express decreased protein levels of E-cadherin (188, 190), in line with cadherin switching from E-cadherin to mesenchymal markers such as N-cadherin (191), fibronectin-1, vimentin, and cadherin-11. Indeed, vimentin, fibronectin-1, and cadherin-11 have been identified in keloids, with overexpression of fibronectin-1 (23, 49). Vimentin, which is commonly expressed in soft tissue tumors, is highly expressed in keloid keratinocytes (190) and associated with changes in shape, motility and adhesion properties during EMT (192). N-cadherin, which is linked to increased tumor cell mobility (193) and progression (194), remains to be studied in keloids. Integrin-linked kinase (ILK), which is normally involved in regulating ECM signaling (195, 196), leads to E-cadherin suppression and promotion of tumor invasiveness (197–200) alongside a fibroblastic phenotype when aberrantly upregulated in EMT. In basal cell carcinoma, ILK expression leads to increased tumor invasiveness and EMT markers through upregulated *Snail*, β-catenin and α-SMA (197). β-catenin is elevated in the nucleus and cytoplasm of cells undergoing EMT. Similarly, a higher level of β-catenin protein activity has been found in keloid keratinocytes compared to normal keratinocytes, and is inversely proportional to E-cadherin expression despite no significant difference at transcription level (140).

Upon injury, epidermal keratinocytes induce dermal fibroblasts to produce keloid matrix features by expressing genes involved in epithelial-to-mesenchymal transition (EMT) (201) and upregulating the expression of inflammatory mediators such as COX1 and COX2 in keloid fibroblasts, endothelial cells, and inflammatory cells (202). The fibroblast-like phenotype in these keloid keratinocytes is potentially perpetuated by the local hypoxic environment in keloids which increase the invasiveness of these keratinocytes, thereby leading to excessive keloid growth (202).

MicroRNAs are known to facilitate oncogenesis and metastasis by regulating post-transcriptional and translational gene expression in cell proliferation, EMT and cancer stem cells (203, 204). This may explain several observations in keloids, for example, the decreased elastic fiber density in keloids despite normal *elastin* expression coding for elastic fibers (205). MiR-21-5p which mediates *PTEN* in keloids, also contributes to EMT in keloids (188, 206), thereby suggesting EMT phenotype maintenance in keloid keratinocytes through exertion of their stem-cell like effects (206). This is significant, as MiR-21-5p has been claimed to be overexpressed in most cancers and various fibrotic disorders such as those involving the skin, kidneys and cardiopulmonary systems (206, 207).

Additionally, insulin-like growth factor-I receptor, which promotes fibroblast invasiveness, is highly upregulated in keloid fibroblasts compared to normal fibroblasts (208). Osteopontin is a major cytokine, which promotes matricellular interaction, tumor progression, angiogenesis, and resistance to apoptosis in malignancies (120). It is expressed in keloids, skin, and mesenchymal tumors. S100 influences the chondroid metaplasia of fibroblasts to fibrocartilage cells (209) and is expressed in desmoplastic melanoma (160), however it has only been found in low levels in keloids (132).

### Resisting Cell Death

Keloid myofibroblasts are thought to sustain extended “healing” by eluding apoptosis (210) despite prolonged hypoxia. Tumor cells may escape apoptosis through periostin-activated Akt/PKB pathway in hypoxia (211). In keloids, periostin activation is linked with downstream activation of ERK1/2 (182) which is known to regulate the functions of Fra-1 and ZEB1/2 in tumorigenesis (212). There is no accepted consensus on the apoptosis levels in keloids. It was previously thought that keloids elude apoptosis by sustaining lower levels of oxidative stress (213). More recent evidence contradicts this; the discovery of increased ROS (125) and downregulated Nrf2 (214) is consistent with other papers suggesting increased rates of apoptosis in keloids (215, 216). Nrf2 is protective against oxidative stresses and various diseases including cancer (217, 218), whereas ROS is thought to act both as a second messenger in intracellular signaling (130) to facilitate cancer progression and as an apoptotic agent by promoting cell senescence (219). This suggests the possibility of differential response to ROS by different cell populations.

Up to eight apoptosis genes were found to be down-regulated amongst 64 which were studied in keloids, compared to normal skin by Sayah et al. (114). Amongst these, *NIP3* is known pro-apoptotic, induced in response to hypoxia by HIF-1α (115). The distribution of apoptotic cells was mapped and found to be equal across the normal tissues but were fewer and concentrated at the margins of keloid tissues. From a regional perspective, Luo et al. (220) described in detail the heterogeneous nature of the microenvironment and subcellular regions in keloids with regard to apoptosis. Within the keloid tissue itself, apoptotic cells are concentrated in hypercellular areas at the edges and lacking in the collagen-abundant center (221), corroborating with previous findings of *p53* and *Bcl-2* localizing to the same regions (79). The concerted up- and down- regulation of various apoptotic-related genes in keloid tissues suggest an interplay of these factors in the tumorigenic capabilities of keloids.

Keloids express several heat shock proteins (HSPs) which are responsible for increasing cell survivability through interprotein interactions in response to stresses (97, 222). HSP47, which is vital in collagen synthesis and maturation (223), is highly expressed in carcinomas of the head, neck (224), and pancreas (225). HSP90 increases in response to cellular stresses to increase cell survival (97) and is upregulated in keloids (98). Similarly, clusterin is increased in cellular stresses (226, 227), and invasive tumors (228).

A study by Yu et al. (140) found increased expression of Wnt10A, β-catenin, and telomerase in keloids. Increased Wnt signaling in keloids promotes cell growth by minimizing apoptosis through upregulation of β-catenin and telomerase activity (140). In normal cells, β-catenin is inhibited by *Sox9* to promote chondrogenesis (229, 230). It is unclear whether these two molecules are simultaneously upregulated and if so, the effects they have on keloid growth. Telomerase activity, which is linked with disease aggressiveness of bone and soft tissue tumors, is found in 10% of benign and 44% of malignant tumors (231). Keloid precursor cells have been shown to demonstrate telomerase activity which may mediate telomere lengthening by interacting with IL-6 (39). There are reports, however, of decreased telomere length (131) and suppressed telomerase activity in keloids as it is thought that telomerase ceases to function after the initial stages of keloid formation (232). Unsurprisingly, telomere length is negatively correlated with ROS levels (131, 233).

It is postulated that a reciprocal protective relationship exists between tumor cells and their stem cell niche, as shown by the upregulation of various genes and proteins in endothelial cells to increase survivability in response to cytotoxic conditions (234, 235). The role of micro-environmental signals (17, 236) which promote chemokine-related changes in keloid fibroblasts (237) has been investigated recently following similar studies on epigenetics in cancer development (238, 239). Studies on DNA methylation profiles of keloid tissue and healthy skin suggest that DNA methylation may be a key driver in the pathology of keloids. These include the discovery of *DNMT1* in keloid fibroblasts (240) and master regulator genes such as pyroxamide, tributyrin, PRKG2, and PENK in keloid tissue (241–243). Other studies found evidence for histone modification such as upregulated HDAC2 in keloid tissue (244) and expression of non-coding RNAs in keloids (245). These discoveries are significant as they represent known biomarkers of cancer. HSPs such as HSP27, HSP47, and HSP70 are also found to be overexpressed in keloids (246), with HSP27 and HSP70 having links to cancer (247).

### Enabling Replicative Immortality: The Role of a Keloid Stem Cell Niche

Stem cells perform various roles in cutaneous wound healing (248). Distinct populations of several stem or stem-like cells (249) have been identified in keloid tissues, including hematopoietic stem cells, mesenchymal-like stem cells, and keloid progenitor cells (40). The fate of stem cells dictated by signals from the stem cell niche microenvironment (250) represents a tightly regulated process in keloids (251).

A population of keloid precursor stem cells (KPCs) was described in the dermis which harbor the capacity for multi-lineage differentiation and self-renewal, the latter of which may explain the high recurrence rates in keloids (39). Their telomerase activity is higher than normal skin precursor cells but lower than cancers. The significance of this is their correlation with tumor or cancer stem cells (252) which are thought to originate from adult stem cells mutated under the influence of a deregulated stem cell niche, following which they become capable of uncontrolled growth, perpetual self-renewal and multi-lineage differentiation, eventually leading to cancer. They may also have a role in conferring resistance to treatment (253, 254). The role of the cancer stem cell niche is outlined in further detail by Borovski (255).

As part of the wound healing process, orchestrated upregulation and interactions between neutrophils, eosinophils, T-cells, B-cells, macrophages, and mast cells have been demonstrated in keloid tissue, and more recently along with keloid-associated lymphoid tissue (KALT) (41, 256). Within KALT, primitive cells within the sub-epidermal micro-vessel endothelium have been recently found to express embryonic stem cell (ESC) markers Oct4, Sox2, pSTAT3, and Nanog (112), suggesting a possible relationship between the immunogenicity and presence of stem cells in keloids.

## Responsiveness to Cancer Treatments

High quality evidence on keloid treatment is limited (257, 258). Larger keloids often require surgical excision (259) which carry recurrence rates of up to 100% as monotherapy (8). Radiation, which is another long-established modality of treatment, has been shown to produce varying results in the literature. Primary radiation given to a sample of 84 unresectable keloids over 5 weeks resulted in 97% significant regression in the course of 18 months (260). Another study found that post-operative radiotherapy in the form of beta radiation at 400cGy twice a week totaling up to 16Gy was found to result in flattened scars in 67% of patients, but not without side effects such as pain and atrophy, and carries a recurrence rate of 21.2% (258, 261). Brachytherapy has also yielded favorable results, with a recurrence rate of 3.1% at 33.6 months in a recent study (262). Combined with surgical excision and application of platelet-rich plasma, post-operative superficial radiotherapy up to three fractions has been demonstrated to result in low disease recurrence (263).

Chemotherapy and targeted therapy agents have also shown promising results. Sorafenib, a tyrosine kinase inhibitor, has been found to be effective in suppressing keloid activity by targeting the TGF-β/Smad and MAPK/ERK pathways which are involved in keloid fibroblast growth and cell cycle processes (264). A recent systematic review identified post-excision 5-fluorouracil in combination with triamcinolone acetonide as a favorable treatment with up to 92% non-recurrence (265) in what is termed as “combination therapy.” In comparison, monotherapy using intra-lesional corticosteroids, the first-line treatment for keloids (8) has been shown to yield positive results in only 77% of patients (266). The recurrence rates in triple therapy incorporating surgical excision, intra-lesional steroids, and silicone sheet have been reported at 4.6% (263), 9.1% (267), and 12.5% (268). Keloids are also responsive to recombinant adenovirus-mediated double suicide gene therapy using CDglyTK which is used in cancer (269).

The use of photodynamic therapy (PDT) in keloids led to the suppression of blood flow and collagen levels alongside improved tissue flexibility (270). This is a cancer treatment used in Bowen's, basal cell carcinoma and actinic keratosis, thought to suppress fibroproliferation through protoporphyrin 9 (PpIX)-mediated activation of ROS, which is in turn cytotoxic (271, 272). Although keloids are not metastatic, their responsiveness to multimodal cancer treatment suggests similar underlying disease mechanisms with tumorigenesis.

## Conclusion

The idea that keloids behave like non-malignant locally aggressive cutaneous cancers is not new and this is evident in both its phenotypical and genetic properties. The biomarker expression profile in these diseases highlights the striking parallels between keloids and both benign and malignant mesenchymal tumors across transcriptional, translational, cellular, and tissue levels. Keloids also exhibit characteristics displayed by cancer cells to some degree, in particular the Warburg effect which confers increased survival to keloid cells in hypoxia. Furthermore, signaling pathways common to these diseases have been found to mold the matrix composition of keloids with the inclusion of chondrogenic signatures. This, in turn, is perpetuated by key cells such as melanocytes, keratinocytes, fibroblasts, and those from the fibrocytic family, which participate in EMT and possess stem cell-like properties. A keloid stem cell niche has also been postulated to support this. Many important tumor-related factors, which have been shown to contribute to the overall pathogenesis by influencing cellular processes such as apoptosis. To this point in time, the use of cancer treatments in keloids has shown encouraging results, further diminishing the fine line between keloids and cancerous tumors. Taken together, this poses many questions in relation to keloid pathobiology which need to be answered, in order to understand how the cascade encompassing prolonged, dysregulated wound healing culminates in the cancer-like or quasi-neoplastic processes which result in keloid formation, progression, persistence and recurrence. This is vital due to the implications it may have for the future therapy and further investigative research of this elusive disease and those with which it shares similar properties.

## Author Contributions

The review article was originally conceived and structured by AB. ST performed literature search and wrote the article. NK contributed by reading and editing. AB edited the overall article.

### Conflict of Interest Statement

The authors declare that the research was conducted in the absence of any commercial or financial relationships that could be construed as a potential conflict of interest.
